# Organization of self-advantageous niche by neural stem/progenitor cells during development via autocrine VEGF-A under hypoxia

**DOI:** 10.1186/s41232-022-00254-2

**Published:** 2023-02-01

**Authors:** Taichi Kashiwagi, Yuuki Takazawa, Tetsushi Kagawa, Tetsuya Taga

**Affiliations:** 1 Department of Histology and Neuroanatomy, Tokyo Medical University, Tokyo, 160-8402 Japan; 2 Department of Stem Cell Regulation, Medical Research Institute, Tokyo Medical and Dental University (TMDU), Tokyo, 113-8510 Japan

**Keywords:** neural stem/progenitor cells, NSPC niche, self-organization, autocrine, hypoxia, vascular endothelial growth factor

## Abstract

**Background:**

Tissue stem cells are confined within a special microenvironment called niche. Stem cells in such a niche are supplied with nutrients and contacted by other cells to maintain their characters and also to keep or expand their population size. Besides, oxygen concentration is a key factor for stem cell niche. Adult neural stem/progenitor cells (NSPCs) are known to reside in a hypoxic niche. Oxygen concentration levels are lower in fetal organs including brain than maternal organs. However, how fetal NSPCs adapt to the hypoxic environment during brain development, particularly before pial and periventricular vessels start to invade the telencephalon, has not fully been elucidated.

**Methods:**

NSPCs were prepared from cerebral cortices of embryonic day (E) 11.5 or E14.5 mouse embryos and were enriched by 4-day incubation with FGF2. To evaluate NSPC numbers, neurosphere formation assay was performed. Sparsely plated NSPCs were cultured to form neurospheres under the hypoxic (1% O_2_) or normoxic condition. VEGF-A secreted from NSPCs in the culture medium was measured by ELISA. VEGF-A expression and Hif-1a in the developing brain was investigated by in situ hybridization and immunohistochemistry.

**Results:**

Here we show that neurosphere formation of embryonic NSPCs is dramatically increased under hypoxia compared to normoxia. *Vegf-A* gene expression and its protein secretion were both up-regulated in the NSPCs under hypoxia. Either recombinant VEGF-A or conditioned medium of the hypoxic NSPC culture enhanced the neurosphere forming ability of normoxic NSPCs, which was attenuated by a VEGF-A signaling inhibitor. Furthermore, in the developing brain, VEGF-A was strongly expressed in the VZ where NSPCs are confined.

**Conclusions:**

We show that NSPCs secret VEGF-A in an autocrine fashion to efficiently maintain themselves under hypoxic developmental environment. Our results suggest that NSPCs have adaptive potential to respond to hypoxia to organize self-advantageous niche involving VEGF-A when the vascular system is immature.

**Supplementary Information:**

The online version contains supplementary material available at 10.1186/s41232-022-00254-2.

## Background

Tissue stem cells are located in special microenvironment called niche [1]. Stem cell niche is known to provide optimal conditions to tissue stem cells for maintenance of multipotency and proliferative capacity, for instance via secretion of soluble factors and through cell-cell contact. Oxygen concentration is one of the key factors for stem cell niche. It is often the case that oxygen concentration is lower in niche than surrounding tissues, but in some cases, such as mesenchymal stem cells (MSCs), MSCs reside near endothelial cells and their environment is relatively high in oxygen concentration, largely attributable to blood vessels [2]. In the hematopoietic stem cell (HSC) niche, bone marrow is more hypoxic compared to other tissues and oxygen concentration is assessed to be less than 1% [3, 4]. In cancer tissues, cancer stem cell (CSC) niche provides appropriate microenvironment for quiescence, self-renewal and differentiation of CSCs. CSC niche is formed apart from blood vessels and cancer cells around CSCs rapidly proliferate, consuming O_2_, and consequently CSCs are under hypoxic conditions [2]. In glioma, peri-vascular and peri-arteriolar niches are reported, but due to vascular dysfunction and inconsistent oxygen delivery, these niches are thought to be hypoxic [5, 6]. Thus, hypoxic niches are considered to harbor stem cells. Under hypoxic conditions, stem cells can avoid from toxicity of reactive oxygen species that are generated near the end of the metabolic series of reactions in the electron transport chain. Furthermore, hypoxic conditions up-regulate expression of stem cell maintenance proteins such as Oct4 and those involved in the Notch signaling pathway via hypoxia-inducible factors (HIFs) [7, 8]. Indeed, low oxygen concentration prevents differentiation of embryonic stem (ES) cells [9], and induced pluripotent stem cells (iPSCs) are more efficiently generated under hypoxic conditions [10]. During embryogenesis, oxygen partial pressure (pO_2_) in placenta is lower than maternal tissues even oxygenated blood is supplied from mother [11]. In mammals, the surface of the brain is higher in oxygen concentration than inside [12]. Neural stem cells (NSCs) are confined to the ventricular zone (VZ) of the brain at the embryonic stage and migrate to pial surface as NSCs differentiate to neurons [13]. Furthermore, vascular network is incomplete during brain development. Therefore, it is speculated that neural stem/progenitor cells (NSPCs) in the developing brain are located in the more hypoxic niche to maintain their population with stem cell properties. In the subventricular zone (SVZ) of lateral ventricle (LV) and subgranular zone (SGZ) of hippocampal dentate gyrus (DG), where neurogenesis occurs even in adult brain, NSPCs contact or are located near various cell types and structures, such as endothelial cells and pericytes of blood vessels, astrocytes, neurons, microglia, ependymal cells and choroid plexus that form NSPC niche and contribute to NSPC maintenance [14]. Since cell differentiation and diversity are poor in the developing brain, embryonic NSPCs are not able to receive sufficient assistance from these cells and structures. In addition, the VZ where NSPCs are confined is under hypoxic conditions partly because vascular network is incomplete. Until now, NSPC niche components, particularly those under hypoxia, are not fully elucidated in the developing brain. We used the enriched NSPC culture and the neurosphere formation assay which reflects the presence of NSPCs to elucidate contribution of hypoxic conditions to NSPC maintenance [15, 16]. Our current study strongly suggest the organization of self-advantageous niche by NSPCs via autocrinely produced vascular endothelial growth factor-A (VEGF-A) in response to hypoxia.

## Methods

### Animals

Pregnant ICR mice were purchased from Japan SLC, Inc. All animal experiments were approved by the institutional Animal Care and Use Committee of Tokyo Medical and Dental University (approval number; 0110267C, 0110299C).

### NSPC Culture

Cortical cells were isolated from embryonic day (E) 11.5 and E14.5 ICR mouse cerebral cortex. In brief, the telencephalons were triturated in Hank’s balanced salt solution (HBSS, Sigma) by mild pipetting. Dissociated cells were cultured in Dulbecco’s modified Eagle medium-F12 (DMEM/F12, ThermoFisher) containing fibroblast growth factor 2 (FGF2, 10 ng/ml, PeproTech), N2 supplement, Antibiotics-Antimycotics solution (100 units/ml of penicillin, 100 μg/ml of streptomycin, and 0.25 μg/ml of Amphotericin B, ThermoFisher) on dishes pre-coated with poly-L-ornithine (Sigma) and fibronectin (O/F-coated dish) (ThermoFisher). N2 supplement contains insulin (25 μg/ml, Sigma), apo-transferrin (100 μg/ml, Sigma), putrescine (16 μg/ml, Sigma), progesterone (20 nM, Sigma), selenite (30 nM, Sigma). To enrich NSPCs, the cells were incubated for 2 or 4 days in the presence of FGF2, then the cells were treated with HBSS and harvested by pipetting [17]. After re-plating, over 90 % of the cells were positive for the NSPCs marker nestin. Cells were cultured at 37 °C under normoxic (95% air, 5% CO_2_ (approximately 20% O_2_)) or hypoxic (1% O_2_, 94% N_2_, 5% CO_2_) conditions. For detection of apoptotic cells, the enriched NSPCs were cultured for one day in the N2 and FGF2 supplemented medium. Then the cells were cultured for 4 days before fixation. For BrdU incorporation assay, 10 μM of BrdU (Sigma) and VEGF-A (10 ng/ml, R & D systems) was administrated into culture medium 12 hrs before fixation.

### Preparation of conditioned medium (CM)

The dissociated cortical cells or the enriched NSPCs isolated from E11.5 or E14.5 cerebral cortex were plated on O/F-coated 60 mm dishes (2 x 10^6^ cells/ml in 5 ml medium). The attached cells were cultured under normoxia or hypoxia for 2 days. The supernatant was harvested and filtered through 0.45 μm pore-size PVDF filter (Millipore) to remove cells and debris. The CM was stored under 4 °C before use.

### Neurosphere assay

To form neurospheres, the cortical cells or enriched NSPCs were cultured at low density (1 x 10^4^ cells/ml in 4 ml medium) for 7 days on 60 mm dishes pre-coated with poly 2-hydroxyethyl methacrylate (poly-HEMA, Sigma) in N2-supplemented DMEM/F12 medium containing FGF2 (10 ng/ml, PeproTech). FGF2 was administrated into culture medium every 2 days in the period of primary neurosphere formation. To investigate the VEGF-A effect on neurosphere formation, VEGF-A (10 ng/ml) and/or SU1498 (700 nM, Merck-Millipore) was administrated in addition to FGF2. To form secondary neurospheres, primary neurospheres were harvested and dissociated by 0.25 % trypsin/PBS with 0.5 % glucose. The dissociated cells were plated in 60 mm dishes and cultured at low density (5 x 10^3^ cells/ml in 4 ml medium) with FGF2 and epidermal growth factor (EGF, 10 ng/ml each, PeproTech) for 7 days. FGF2 and EGF were administrated into culture medium every 2 days in the period of secondary neurosphere formation. Neurospheres of 50 μm diameter or larger were counted. At least three independent dishes were counted. The neurosphere numbers tend to fluctuate even under the same culture condition. Thus, for the benefit of the readers, the actual neurosphere number in each dish is provided in Figure S1, S7, S9 and S14. This probably arose from individual difference in each embryo in each set up experiment. However, the hypoxic effects were consistent in the identical lots. Thus, the ratio of neurosphere numbers to the control was calculated.

### Reverse transcription-PCR

Total RNA was prepared from enriched NSPCs derived from E14 cortices by using Isogen (Nippon gene). First-strand cDNA was synthesized from total RNA by using Superscript III First-Strand Synthesis System (ThermoFisher). The PCR was performed using GoTaq Green Master Mix (Promega) with 21 (*ß-actin*), 24 (*Vegf-A*) and 28 (*Vegfr-1*, *Vegfr-2*) cycles of denaturation at 94 °C for 20 sec, annealing at 60 °C for 30 sec, and extension at 72 °C for 45 sec. Specific primers were the following; *Vegf-A* sense primer, 5’- caggctgctgtaacgatgaagc -3’; antisense primer, 5’- caccgccttggcttgtcaca -3’; *Vegfr1* sense primer, 5’- cggaaggaagacagctcatc -3'; antisense primer, 5’- catacacatgcacggaggtg -3', *Vegfr2* sense primer, 5’- ggtctttcggtgtgttgctc -3'; antisense primer, 5’- tctgtctggctgtcatctgg -3', ß-actin sense primer, 5’- ccagggtgtgatggtgggaa -3'; antisense primer, 5’- cagcctggatggctacgtaca -3'.

### In situ hybridization

RNA probes were synthesized from 0.5 kbp of *Vegf-A* cDNAs (sequence was referred to NM_001025250.3). Briefly, *Vegf-A* cDNAs were amplified by PCR from mouse cDNAs by using the identical primer set used for reverse transcription-PCR. *Vegf-A* cDNAs were cloned into pBluescript II KS(+). Digoxigenin (DIG)-labeled antisense RNA probes of *Vegf-A* were synthesized by DIG RNA labeling kit (Roche). For in situ hybridization, RNA probes were hybridized at 65 °C for 16 hrs, and then brain sections were incubated with alkaline phosphatase-conjugated anti-DIG antibody (Roche) overnight at 4 °C after blocking with Brocking Reagent (Roche). Probes were colored by 50 μg/ml of p-nitroblue tetrazolium chloride (NBT, Roche) and 175 μg/ml of 5-bromo-4-chloro-3-indolyl phosphate (BCIP, Roche).

### Immunostaining

Embryonic brains were fixed with 4 % paraformaldehyde overnight at 4 °C. The cells were fixed with 4 % paraformaldehyde for 20 minutes at room temperature. For immunostaining and immunohistochemistry, cells or tissues were blocked with phosphate-buffered saline (PBS) containing 10 % serum and 0.1 % Triton X-100 for over 30 minutes and were incubated overnight at 4 °C with primary antibodies. For staining with anti-BrdU antibody, samples were treated with 2N HCl for 30 min at 37 °C to denature DNA after fixation. The samples were examined by epifluorescence after 60 min incubation with secondary antibodies at room temperature and washing. Cell nuclei were counterstained with Hoechst 33258 (Nacalai Tesque). Fluorescent images were obtained using IX70 fluorescence microscope (Olympus) and 700 confocal laser scanning microscope (Carl Zeiss). The following primary antibodies were used: rat anti-BrdU (1:500, abcam, ab6326), rabbit anti-ß-tublin III (1:1000, BioLegend, PRB-435P), mouse anti-nestin (1:1000, BD Bioscience, 556309), rabbit anti-active Caspase-3 (1:1000, BD Bioscience, 559565), rabbit anti-Hif-1a (1:500, Nobus Biologicals, NB100-479), rabbit anti-GFAP (1:1000, DAKO Z0334), goat anti-Sox2 (1:500, R&D Systems, AF2018), mouse anti-NeuN clone A60 (1:500, Merck, MAB377). The following secondary antibodies were used: Alexa Fluor 488-conjugated goat anti-mouse IgG (1:1000, ThermoFisher), Alexa Fluor 488-conjugated goat anti-rabbit IgG (1:1000, ThermoFisher), Alexa Fluor 488-conjugated donkey anti-mouse IgG (1:1000, ThermoFisher), Alexa Fluor 546-conjugated goat anti-rabbit IgG (1:1000, ThermoFisher), Alexa Fluor 546-conjugated goat anti-rat IgG (1:1000, ThermoFisher), Alexa Fluor 555-conjugated donkey anti-rabbit IgG (1:1000, ThermoFisher), Alexa Fluor 647-conjugated donkey anti-goat IgG (1:1000, ThermoFisher).

### ELISA

VEGF-A content of culture medium was quantitated by using Mouse VEGF-A ELISA kit (R&D Systems). 450 nm wavelength of the reaction solution was measured by iMARK microplate leader (BioRad).

### Statistical analysis

Statistical differences were determined by Welch’s two-sided t-test unless specifically indicated. In Figure 3A and Figure S10, one-sided test was also performed, since VEGF-A is supposed to exhibit unidirectional effect, which is enhancement but not suppression of neurosphere formation, in the context of previous studies reporting contribution of VEGF-A to NSC proliferation and survival [18]. The asterisks indicate statistically significant (*, *p* < 0.05; **, *p* < 0.01; ***, *p* < 0.001)

## Results

### Neurosphere formation of embryonic NSPCs is increased under the hypoxic condition.

**Fig. 1:**
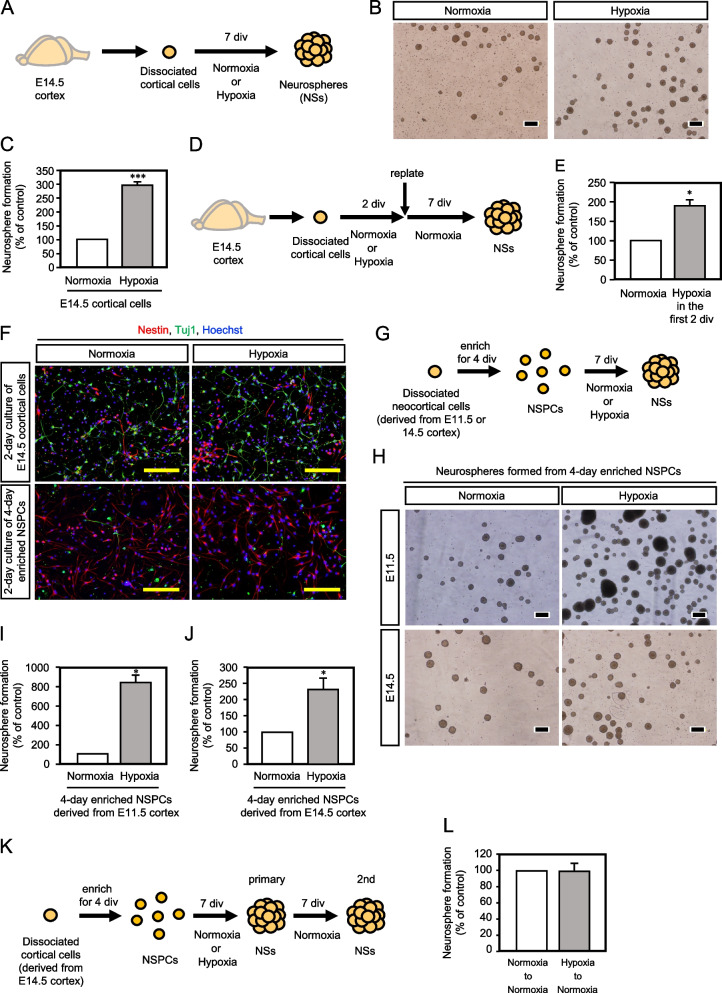
Neurosphere formation is enhanced under hypoxic condition compared to normoxic condition. **A** Experimental procedure of (B)-(C). **B** Microphotographs of neurospheres formed from E14.5 cortical cells under the normoxic or the hypoxic condition. Scale bar = 200 μm. **C** Quantification of neurosphere formation in E14.5 cortical cells under the normoxic or the hypoxic condition. The data is shown relative value of neurosphere numbers of hypoxic condition based on control value 100. The neurosphere number under the hypoxic condition was increased five times compared to under the normoxic condition (mean ± SEM, *n* = 5, *** *p* = 0.0000642). **D** Experimental procedure of (E). Cortical cells isolated from E14.5 brain were pre-incubated for 2 days under the normoxic or the hypoxic condition before the neurosphere formation period of the normal conventional condition. **E** Quantification of neurospheres after pretreatment of hypoxia in the first 2 div (mean ± SEM, *n* = 3, * *p* = 0.044). **F** Immunofluorescence staining of E14.5 mouse cortical cells (upper panels) and enriched NSPCs (lower panels). The cortical cells were cultured for 2 days in the presence of FGF2 under the normoxic or the hypoxic condition after the cells were isolated from E14.5 cortex (see the CM preparation in Fig. 2A). The enriched NSPCs were obtained by 2-day culture in the presence of FGF2 under the normoxic and the hypoxic conditions after 4-day culture of cortical cells under the conventional normal condition and replating (see the CM preparation in Fig. 2C). Not only nestin-positive NSPCs but also Tuj1-positive neurons were observed in the cortical cell culture, while most of the cells were NSPCs after the enrichment. Scale bar = 100 μm. **G** Experimental procedure of (H). **H** Neurospheres were formed from enriched E11.5 brain derived and E14.5 brain derived NSPCs. Scale bar = 200 μm. **I**, **J** Quantification of E11.5 NSPCs derived (I, mean ± SEM, *n* = 3, * *p* = 0.0120) and E14.5 NSPCs derived neurospheres (J, mean ± SEM, *n* = 4, * *p* = 0.0432). **K** Experimental procedure of (L). E14.5 enriched NSPCs were cultured for 7 days to form primary neurospheres under the normoxic or the hypoxic condition. Then, the primary neurospheres were scattered and re-plated to form secondary neurospheres under the normoxic condition. **L** Quantification of the secondary neurospheres (mean ± SEM, *n* = 3)

To investigate the effect of hypoxia on embryonic NSPC characteristics, neurpsphere formation assay which reflects the presence of NSCs was performed under normoxia and hypoxia. Cortical cells were isolated from E11.5 or E14.5 mouse forebrain and plated in non-adhesive dishes at a clonal density (< 10000 cells/ml) [19, 20] to form neurospheres derived from single NSPCs. The cells were cultured for 7 days to form neurospheres under the normal condition (20% O_2_) or the hypoxic condition (1% O_2_) (Fig. 1A). The hypoxic condition increased neurosphere formation of cortical cells isolated from E14.5 brain. Representative photographs of neurospheres are shown in Figure 1B. Quantitative analysis showed that 2.90-fold increase in neurosphere formation was observed under the hypoxia, compared with the normoxia (Fig. 1C, S1A), suggesting that more NSPCs are maintained in hypoxia than normoxia. To confirm this notion, embryonic cortical cells were exposed under the hypoxic condition for 2 days before neurosphere formation period, then neurospheres were formed under the conventional normal condition (Fig. 1D). As shown in Figure 1E and S1B the 2-day hypoxia-exposed cells formed 1.80-fold larger number of neurospheres compared to the control, suggesting that hypoxia exposed NSPCs had been already expanded more at the start point of the neurosphere formation culture compared to the normoxia control. To determine whether this phenomenon is NSPC-autonomous or is dependent on the surrounding cells, cortical cells isolated from E14.5 brains were stained for NSPC marker nestin and neuronal marker Tuj1 after 2-day culture under the normoxia or hypoxia. As shown in upper panels of Figure 1F, nestin-positive NSPCs as well as neurons were observed. To exclude the possibility that factors secreted from neurons might contribute to NSPC stemness maintenance, we enriched NSPCs for 4 days in the presence of FGF2 and then the NSPCs were replated by pipetting to remove neurons before neurosphere assay. Four days after enrichment, the cells were replated, then the cells were further cultured for 2 days under the normoxia or hypoxia. Approximately 30 % each of nestin-positive cells and Tuj1-positive cells were observed after the 2-day culture of cortical cells, while approximately 80 % nestin-positive cells and approximate 3 % Tuj1-positive cells were observed after the 4-day enrichment culture and the replating (Fig. 1F, S2). In addition, GFAP-positive astrocytes were hardly observed in the conditions unless GFAP-expression was induced by LIF and BMP2 administration [21] (Fig. S3). These results suggest dramatic enrichment of NSPCs by the 4-day culture and the replating as compared with the cortical cell culture. We also used NSPCs isolated from brains at E11.5 that is a stage when the vascular network is more immature than at E14.5. Using enriched NSPCs from E11.5 and E14.5 cortical cells, neurosphere formation assay was performed under the normal or hypoxic condition (Fig. 1G). Hypoxia increased neurosphere formation of enriched NSPCs isolated from both stages (Fig. 1H for representative photographs, 1I, S1C; 1J, S1D for quantification). The distribution of neurosphere size appears to be not much different between normoxia and hypoxia (Fig. S4A). Of note, the hypoxic condition strongly increased the number of neurospheres of diameters of both smaller and larger than 50 μm (Fig. S4B). In this assay, we set the cut off value of the neurosphere diameter at 50 μm to exclude spheres and clusters derived probably from non-NSPCs [20] so that the assay appropriately detects the effect of the hypoxic condition on NSPCs. These data suggest that hypoxia most likely acts to enhance stemness of NSPC in a cell-autonomous manner either by a cell-intrinsic mechanism or through an autocrine fashion. Of note, increase rate of E11.5 neurosphere formation (8.22-fold) was higher than that of the E14.5 one (2.09-fold), suggesting that younger NSPCs are accommodated to hypoxic condition, possibly due to immature vascular network. With the enriched NSPCs, secondary neurosphere assay was performed, in which the NSPCs were subjected to primary neurosphere culture under normoxia and hypoxia, and then to secondary neurosphere culture under the conventional normal condition (Fig. 1K). Statistically equivalent numbers of neurospheres were observed in the secondary assay (Fig. 1L, S1E). Since NSPCs can maintain undifferentiated status for long term in neurosphere state [22], the secondary neurosphere assay is ordinary performed to evaluate the presence of NSPCs, or in other words, the self-renewal capacity of the cultured NSPCs. Therefore, since the almost equivalent numbers of secondary neurospheres were detected in Figure 1L, it is indicated that the dramatic increase in the neurosphere numbers under hypoxia in Figure 1C, E, I, and J highly probably reflects the enhancing effect of hypoxia on NSPC stemness. In agreement with this notion, the hypoxic condition significantly hindered NSPCs to differentiate into NeuN-positive neurons compared to the normoxic condition (Fig. S5). These results support the idea that hypoxic condition contributes to maintenance of undifferentiated status of NSPCs.

### NSPCs secret autocrine factor(s) for their maintenance under hypoxia.

**Fig. 2.**
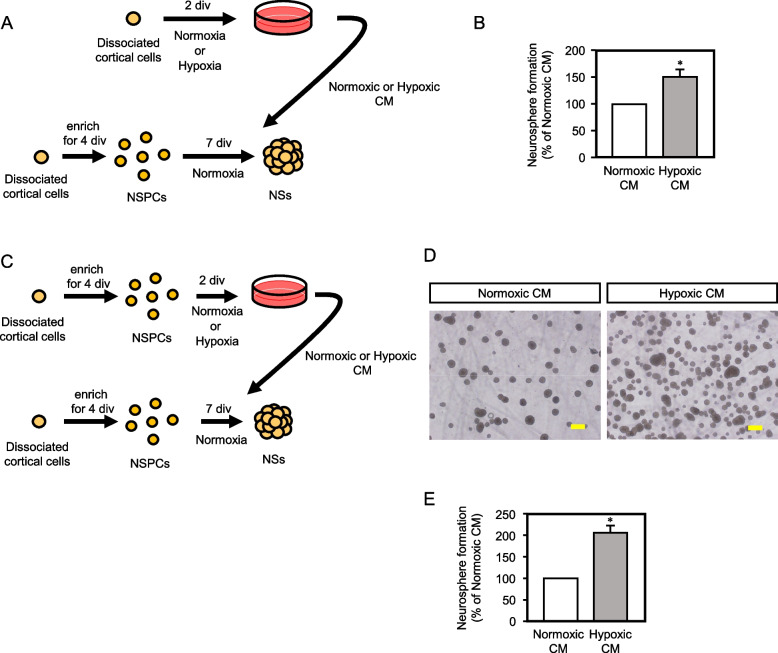
NSPCs secret factor(s) enhancing neurosphere formation in an autocrine manner under hypoxic condition. **A** Experimental procedure of (B). The CM was harvested from E14.5 cortical cell culture for 2 days under the normoxic or the hypoxic condition. Neurosphere formation derived from E14.5 enriched NSPCs was performed under the conventional normal condition with CM. **B** Quantification of the neurospheres formed under the CM (mean ± SEM, *n* = 4, * *p* = 0.0355). **C** Experimental procedure of (D)-(E). The CM was harvested from enriched NSPC culture for 2 days under the normoxic or the hypoxic condition. Enriched NSPCs was cultured under the CM from the normal O_2_ atmosphere. **D** Microphotographs of neurospheres formed under the CM from the normoxic or the hypoxic enriched NSPC culture. Scale bar = 200 μm. **E** Quantification of the neurospheres formed under the normoxic or the hypoxic CM (mean ± SEM, *n* = 3, * *p* = 0.0270).

Next, to examine whether the NSPC-autonomous increase in neurosphere formation under the hypoxic condition is mediated by a cell-intrinsic mechanism or through an autocrine fashion, we first tested CM of E14.5 cortical cell culture under hypoxia. As illustrated in Figure 2A, enriched E14.5 NSPCs were subjected to 7-day-neurosphere cultures with the normoxic- or the hypoxic CM. The hypoxic CM led to the 1.50-fold increase in the neurosphere number (Fig. 2B, S6, S7A). This effect was not observed by the medium treated by hypoxia for 2 days in the absence of cells (Fig. S8), excluding the possibility that hypoxia-treated medium component(s) enhanced neurosphere formation. Because not only NSPCs but also neurons are present in the cortical cell culture (Fig. 1F, S2A), we then prepared CM of enriched NSPCs that were cultured under the normoxia or the hypoxia for 2 days (Fig. 2C, S2B). The ratio of NSPCs and that of neurons to the total cells were comparable and not statistically different between the normoxic and the hypoxic cultures. As shown in Figure 2D, E, and S7B the CM prepared from the enriched NSPC hypoxic culture increased the neurosphere number by 2.07-fold compared to the normoxic control. Of note, a 1.50-fold increase in the neurosphere number by the CM prepared from the cortical cell culture that included approximate 70 % of the cells other than NSPCs were observed, while the CM prepared from the enriched NSPC culture that included approximate 80 % of the NSPCs led to the 2.07-fold increase. Therefore, these results strongly suggest that, under the hypoxic condition, factor(s) which enhance(s) neurosphere formation is mainly secreted from NSPCs. Taken together, NSPCs under hypoxia adapt by secreting effective factor(s) to maintain themselves.

### VEGF-A secreted from NSPCs in hypoxia contributes to their maintenance.

**Fig. 3.**
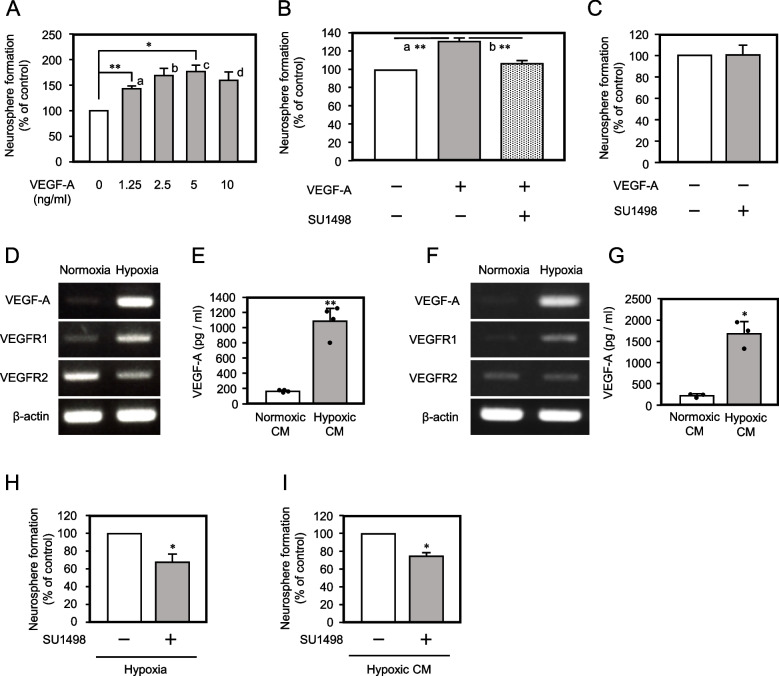
VEGF-A is secreted from NSPCs and enhances neurosphere formation under hypoxic condition. **A** Quantification of neurospheres from NSPCs by administration of VEGF-A (mean ± SEM, *n* = 3, ** *p* = 0.00754, * *p* = 0.0436, (^a^ *p* = 0.00377, ^b^ *p* = 0.0305, ^c^ *p* = 0.0218, ^d^ *p* = 0.0473 to the control in the case of one-sided test)). **B** Quantification of neurospheres from NSPCs by administration of 10 ng/ml of VEGF-A and 700 nM of VEGFR2 inhibitor, SU1498 (mean ± SEM, *n* = 3, ^a^ *p* = 0.00555, ^b^ *p* = 0.00695). The equivalent volume of DMSO which was used as solvent of SU1498 was administrated in the control. **C** Neurosphere formation from enriched E14.5 NSPCs in the presence of SU1498 (700 nM) under the normoxic condition. **D** VEGF-A gene expression in NSPCs isolated from E11.5 brain under the hypoxic condition was analyzed by semi-quantitative reverse transcription-PCR. The gel images were cropped and the full-length gel images were shown in Fig. S12A. **E** Measurement of VEGF-A in the E11.5 NSPC culture under the normoxic or the hypoxic condition by ELISA (mean ± SEM, *n* = 4, ** *p* = 0.002858). Each black dot indicates the VEGF-A values in each sample. **F** VEGF-A gene expression in NSPCs isolated from E14.5 brain under the hypoxic condition was analyzed by semi-quantitative reverse transcription-PCR. The gel images were cropped and the full-length gel images were shown in Fig. S12B, C. The gel image of VEGF-A was inverted horizontally. **G** Measurement of VEGF-A in the E14.5 NSPC culture under the normoxic or the hypoxic conditions by ELISA (mean ± SEM, *n* = 3, * *p* = 0.01448). **H** Quantification of neurospheres of NSPCs formed in the hypoxic neurosphere formation culture supplemented with or without SU1498 (mean ± SEM, *n* = 4, * *p* = 0.0482). **I** Quantification of neurospheres of NSPCs formed in the conventional normoxic neurosphere formation culture with the hypoxic NSPC CM, supplemented with or without SU1498 (mean ± SEM, *n* = 4, * *p* = 0.0107)

We focused on VEGF as a candidate to maintain NSPCs since the *Vegf* gene is an important transcriptional target of HIF-1 (hypoxia inducible factor 1) alpha (Hif-1a) that is stabilized under hypoxic conditions [23]. Thus, various concentrations of VEGF-A were added to the enriched E14.5 NSPC neurosphere formation culture. Neurosphere formation was increased by VEGF-A in a dose dependent manner, which reached a plateau at 2.5 ng/ml (Fig. 3A, S9A). Increase in neurosphere number by administration of VEGF-A was also observed with enriched E11.5 NSPCs (Fig. S10). 0.7 μM of SU1498, a selective inhibitor of VEGF receptor 2 (VEGFR2) tyrosine kinase efficiently diminished the increase in the neurosphere number by VEGF-A (Fig. 3B, S9B, S11). SU1498 did not affect neurosphere number under the normoxic condition (Fig. 3C, S9C), suggesting that increase in neurosphere formation is due to up-regulation of VEGF-A signaling by VEGF-A secretion from NSPCs.

Next, to investigate whether *Vegf-A* expression is up-regulated under the hypoxic condition, 4-day enriched NSPCs were incubated under the hypoxic condition for 6 hrs and *Vegf-A* mRNA expression was analyzed by RT-PCR. *Vegf-A* expression was considerably up-regulated under the hypoxic condition in both NSPCs isolated from E11.5 and 14.5 cortex (Fig. 3D. F). *Vegfr1* expression was also up-regulated under the hypoxic condition, but *Vegfr2* was not (Fig. 3D, F). To confirm these results at the protein level, VEGF-A protein secreted from NSPCs into the culture medium was determined by enzyme linked immunosorbent assay (ELISA). After 2-day incubation, the VEGF-A protein level was 6.65-fold upregulated in the CM of the hypoxic culture of enriched NSPCs from E11.5 cortex (Fig. 3E; Normoxic CM, 165 ± 3.65 pg/ml; Hypoxic CM, 1098 ± 127 pg/ml) and 9.12-fold upregulated in the CM from E14.5 NSPC culture (Fig. 3G; Normoxic CM, 184 ± 32.6 pg/ml; Hypoxic CM, 1678 ± 194 pg/ml). Of note, the VEGF-A protein level in the E14.5 NSPC culture medium under the hypoxic condition tended to be higher than that in the E11.5 NSPC culture medium under the hypoxic condition, although statistical difference was not observed (*p* = 0.07434, Fig. 3E, G, S13). In contrast, the increase rate of neurosphere formation under the hypoxic condition compared to the normoxic condition was significantly and much higher in E11.5 derived enriched NSPCs compared to E14 (Increase rate was 8.37 ± 0.667 in E11.5 NSPC culture (Fig. 1I) and 2.34 ± 0.344 in E14.5 (Fig. 1J), which was statistically significant (*p* = 0.00734)). Therefore, responsiveness to VEGF-A may depend on the developmental stages, or factor(s) in addition to VEGF-A may also contribute to NSPC maintenance. Next, to examine VEGF-A protein secreted from NSPCs contributes to NSPC maintenance, neurosphere assay was performed under the hypoxic condition or the hypoxic CM, supplemented with or without the VEGFR-2 inhibitor SU1498 [24]. Neurosphere number was decreased by SU1498 under the hypoxic sphere-forming culture condition (Fig. 3H and S9D) and also under the normoxic sphere-forming culture condition with hypoxic CM (Fig. 3I and S9E). Together, these results suggest that at least VEGF-A is one of the factors that contributes to NSPC maintenance in an autocrine manner under the hypoxic condition.

### Younger NSPCs secret more effective factors under hypoxic condition.

**Fig. 4.**
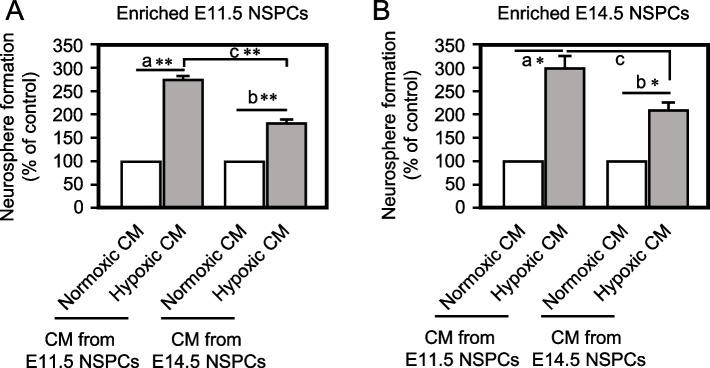
Hypoxic CM from younger NSPCs effectively enhance neurosphere formation. **A** Neurosphere assay of enriched E11.5 NSPCs under the normoxic or hypoxic CM prepared from enriched E11.5 or E14.5 NSPCs. (mean ± SEM, *n* = 3, ^a^ *p* = 0.00537, ^b^ *p* = 0.00983, ^c^ *p* = 0.00653). **B** Neurosphere assay of enriched E14.5 NSPCs under the normoxic or hypoxic CM prepared from enriched E11.5 or E14.5 NSPCs. (mean ± SEM, *n* = 3, ^a^ *p* = 0.0246, ^b^ *p* = 0.0262, ^c^ *p* = 0.0847).

As shown earlier in this article, neurosphere formation under the hypoxic condition was increased to a greater extent in NSPCs of E11.5 than E14.5 (Fig. 1I, J; 6.75-fold and 2.09-fold), suggesting that younger NSPCs secret more effective factor(s) and/or have greater ability to respond to such factor(s) higher than older NSPCs. To investigate this hypothesis, neurospheres were formed from E11.5 or E14.5 cortex-derived enriched NSPCs under the CM collected from cultures of enriched E11.5 or E14.5 NSPCs. In the case of neurospheres formed from E11.5 cortex-derived enriched NSPCs (Fig. 4A, S14A), the CM from hypoxic-culture of E11.5 cortex-derived enriched NSPCs more dramatically increased neurospheres than that from enriched E14.5 NSPCs. Likewise, in the neurospheres derived from E14.5 enriched NSPCs (Fig. 4B, S14B), a similar tendency was observed in which the hypoxic CM collected from E11.5 cortex-derived enriched NSPCs was more effective than that of E14.5, but it was not statistically different. These results suggest that younger NSPCs secret more effective factor(s) under the hypoxic condition for the maintenance of themselves effectively.

### VEGF-A suppresses NSPC apoptosis and induces their growth

**Fig. 5.**
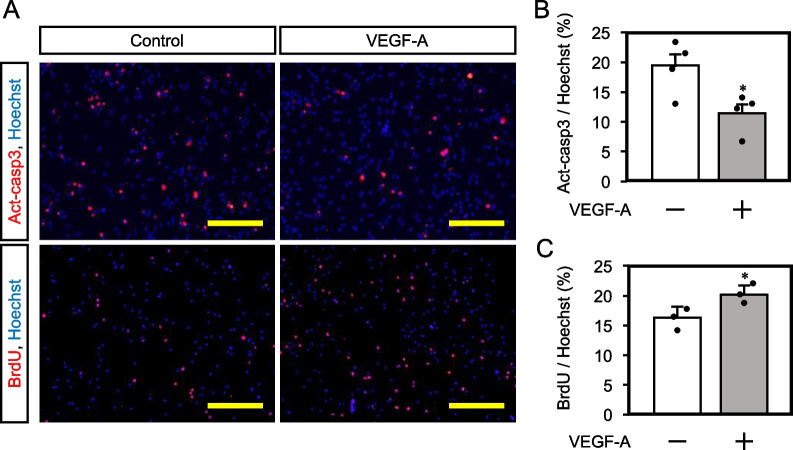
VEGF-A suppresses NSPC apoptosis while promotes NSPC growth. **A** Detection of apoptotic (upper panels) and proliferated (lower panels) NSPCs derived from E14.5 cortex in the absence or the presence of 10 ng/ml of VEGF-A under the normoxic condition. Scale bar = 100 μm. **B** Quantification of apoptotic NSPCs (mean ± SEM, *n* = 4, * *p* = 0.028). Each black dot indicates the ratio of apoptotic NSPCs in each sample. **C** Quantification of proliferated NSPCs (mean ± SEM, *n* = 3, * *p* = 0.049). Each black dot indicates the ratio of BrdU incorporated NSPCs in each sample.

We next investigated the effect of VEGF-A on apoptosis and proliferation of NSPCs. NSPCs derived from E14.5 cortex were cultured for 4 days in the presence of VEGF-A (10 ng/ml), and active caspase-3 immunoreactivity was analyzed (Fig. 5A). The ratio of the active caspase-positive apoptotic cells was significantly decreased by VEGF-A treatment (Fig. 5B, Caspase-3-positive cell ratio to control, 19.5 ± 1.87; VEGF, 11.6 ± 1.42). Since cells double positive for active caspase-3 and Sox2 (an NSPC marker) were also suppressed by VEGF-A administration (Fig. S15), VEGF-A is suggested to protect NSPCs from apoptosis in the culture condition. Conversely, BrdU uptake was increased after treatment of VEGF-A for 12 hrs (Fig. 5C, BrdU-positive cell ratio to control, 16.3 ± 1.28; VEGF, 20.3 ± 0.779). These results suggest that VEGF-A contributes to NSPC maintenance by anti-apoptotic effect and encouragement of NSPC proliferation.

### VEGF-A and Hif-1a are expressed in the VZ where NSPCs exist in the developing brain

**Fig. 6.**
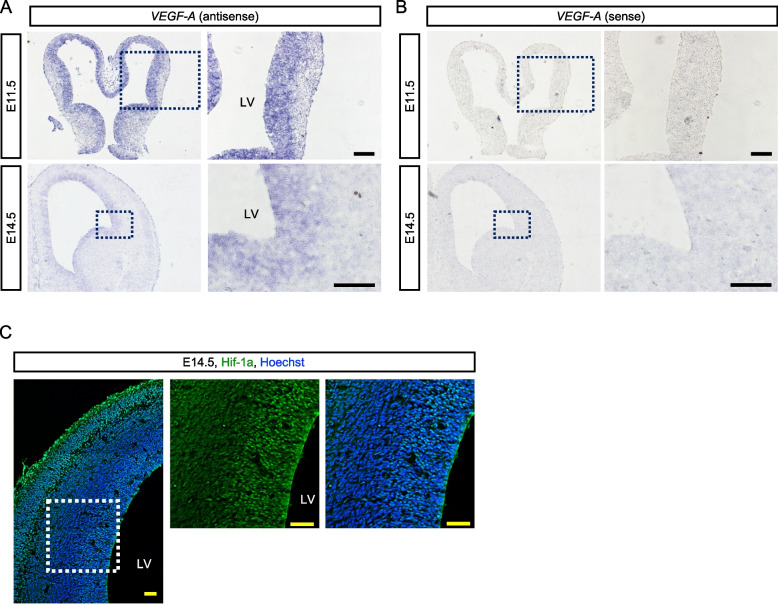
VEGF-A is expressed in the VZ where NSPCs are confined in the developing cortex. **A** Vegf-A mRNA expression in the E11.5 and 14.5 brain was detected by in situ hybridization. Right panels indicate magnification of boxed area. Scale bar = 50 μm. **B** Sense probe was used as negative control. Right panels indicate magnification of boxed area. Scale bar = 50 μm. **C** Immunohistochemistry by anti-Hif-1a antibody in the E14.5 cortex. The center and right panels indicate magnification of boxed area in the left panel. The center panel shows single color image of Hif1-a. Scale bar = 50 μm. LV, lateral ventricle.

Next, to investigate whether VEGF-A is localized in the VZ where NSPCs reside, *Vegf-A* gene expression was detected in the developing brain by in situ hybridization. *Vegf-A* mRNA was observed in the VZ in both E11.5 and 14.5 brain (Fig. 6A, B). In addition, Hif-1a protein that is stabilized under the hypoxic condition and positively regulates *Vegf-A* expression was expressed and localized in nucleus of cells in the VZ where NSPCs are confined and abundantly present at developmental stages (Fig. 6C). Since the VZ is relatively hypoxic [25, 26], these data suggest that the hypoxic condition in the VZ makes NSPCs execute self-maintenance strategies involving VEGF-A expression by stabilized Hif-1a.

## Discussion

NSPC maintenance and fate determination are governed by cell-intrinsic programs like epigenetic regulation as well as extracellular signaling by cytokines and cell-cell contact such as Notch signaling. Adult NSPCs in the DG of hippocampus or the SVZ of lateral ventricle are surrounded by various cell types such as endothelial cells and pericytes of blood vessels, astrocytes, neurons, microglia, and ependymal cells. These cells constitute stem cell niche to maintain and regulate survival and differentiation of NSPCs by direct cell-cell contact or via soluble mediators including nutrients [27–29]. While in the developing brain, VZ where NSPCs are confined is occupied by mostly NSPCs and limited types of cells exist in the developing brain compared to adult brain. Cytokines from cerebrospinal fluid and Notch-Hes signaling by cell-cell contact are crucial factors to regulate NSPC maintenance and fate determination in the developing brain [30, 31]. Notch signaling is up-regulated and collaborates with Hif-1a to promote downstream gene expression such as *Hes* and *Hey* genes under hypoxia as reported in cancer cells [7, 32]. Therefore Notch signaling in hypoxia might also contribute to NSPC maintenance under hypoxia. Our current study provide a novel mechanism of maintenance of NSPCs in the embryonic stages, that is NSPCs themselves constitute stem cell niche under hypoxic conditions through an autocrine fashion by secretion of VEGF-A in the circumstance of embryonic NSPCs surrounded by limited cell types.

In the present study, we propose that embryonic brain forms stem cell niche, which is mainly consist of NSPCs and is different from that of adult brain comprising various cell types and hypoxia contributes to maintenance of NSPCs via VEGF-A secretion from themselves. In two major neurogenic regions of adult brain, i.e., SVZ in the lateral ventricular wall and SGZ in the DG of hippocampus, it was reported that VEGF-A contributes to maintenance of NSPCs and support adult neurogenesis [18, 33]. In this study, CM was from the culture of neurospheres derived from ventricular wall cells of adult brain. Neurospheres are formed from single NSPCs but grown neurospheres are composed of various cell types, not only NSPCs but also neurons and astrocytes. Only 0.16% of NSCs are estimated in neurospheres [34], and another report estimates that 9% of neurosphere forming cells are capable of forming neurospheres in the culture medium with appropriate neurosphere-CM suitable for the maintenance of NSCs [35, 36]. Therefore, in the reports [18, 33], VEGF-A may be secreted from not only adult NSCs but also various cell types, such as neurons and glial cells. In addition, since adult NSPCs are minority population in the SVZ and SGZ, it is uncertain how VEGF-A derived from adult NSPCs contributes to their maintenance. In agreement with this observation, up to two-thirds of VEGF expressed in the DG remains after NSPC-specific knockdown [33]. On the other hand, we harvested the CM from the enriched nestin-positive NSPC culture for short period that is insufficient for differentiation from NSPCs to neurons or astrocytes (see Fig. 1F, S2 for their purity). In addition, astrocyte differentiation is not observed in our NSPC culture condition unless exogenous both BMP2 and LIF are administrated [21]. Thus, our data strongly suggest that VEGF-A in the CM is mainly derived from NSPCs. Besides, our results suggest that an autocrine fashion of VEGF-A secreted from NSPCs is a crucial mechanism for NSPC maintenance in the hypoxic niche at developmental stages. Exogenous VEGF-A induced 1.8-fold increase in the neurosphere number at most (Fig. 3A). Of note, 2.09-fold increase in neurospheres was observed under the hypoxic condition (Fig. 1J), but the number of neurspheres under the hypoxic condition was decreased only by 30% by SU1498 (Fig. 3B). Therefore, it is considered that factors other than VEGF-A may also be involved in NSPC maintenance under hypoxic conditions. Such factors may possibly include chondroitin sulfate proteoglycans secreted from NSPCs [36, 37], and might also include factors derived from residual neurons present in the enriched NSPCs (Fig. 1I, J). FGF2 can be such a factor and partly comprises the hypoxic niche in an autocrine manner, since its expression is observed in the ventricular zone at the developmental stages [38], and a putative regulatory region of Hif-1 exists in the FGF2 promoter [39]. In this relation, HB-EGF secreted from neurons was reported to contribute to the NSPC maintenance [40]. In addition, CM from E11 NSPC culture were more effective on neurosphere formation than that from E14 NSPC culture (Fig. 4), suggesting that younger NSPCs secrete more kinds or amounts of factors contributing to the self-maintenance of NSPCs in addition to VEGF-A under hypoxic conditions. Moreno et al. examined the gene expression profile in the cultured NSCs derived from embryonic mouse cortical cells under the hypoxia [41]. In this study, VEGF-A is dominantly upregulated by the hypoxia among cytokines, suggesting that VEGF-A is the most abundantly expressed cytokine in NSPCs under hypoxic condition. However, Moreno et al. do not mention functional relevance and significance of VEGF-A for NSPC maintenance. Thus, as a cytokine, VEGF-A secreted from NSPCs is most likely involved in NSPC maintenance in an autocrine fashion under hypoxia as we for the first time propose in the current study. As we discussed above, the different effects of CM from E11 NSPCs and E14 NSPCs on neurosphere formation could not be explained in the VEGF-A expression alone, therefore, further studies are necessary to discover secretory factors regulating NSPC self-maintenance under hypoxic conditions.

In this study, we used SU1498 to suppress VEGF signaling. Among VEGFs, VEGF-A mainly plays a role in nervous systems, and VEGFR-1 and VEGFR-2 are receptor for VEGF-A [42]. SU1498 is a specific and potent inhibitor against VEGFR-2 [24, 43]. Based on our results, VEGF signaling via VEGFR-2 is considered to play a crucial role in the maintenance of NSPCs. Supporting our results, VEGF signaling via VEGFR-2 but not via VEGFR-1 promotes growth of glioblastoma cells that possess NSC-like character, and VEGFR-1 has a negative feedback effect on VEGF signaling via VEGFR-2 [44]. Furthermore, VEGFR-1 is not expressed in rat adult NSCs even under hypoxic conditions or presence of VEGF [45]. Taken together, we could not deny contribution of VEGFR-1 to NSPC maintenance because VEGFR-1 expression was observed in cultured NSPCs under the hypoxic condition, but VEGF-A-VEGFR-2 signaling is mainly considered to contribute to maintenance of NSPCs under hypoxic conditions primarily.

Our current in vitro findings provide important concept in understanding development of the brain in consideration of blood vessels. In the developing brain at around E10 pial vessels and periventricular vessels are first constructed and invade into dorsal telencephalon a little before formation of pial vascular plexus. Arterial blood vessels are thought to be originated from subventricular vascular plexus [46, 47], and vasculature of germinal zone is sprouted from it. However, only fine filopodia from subventricular vascular plexus vasculature extended to germinal zone in hindbrain and cortex at embryonic stages [26, 48]. Furthermore, oxygen consumption of actively dividing NSPCs is thought to be greater than that of postmitotic cells. In agreement with these notions, Hif-1a, stabilized under hypoxic conditions, is strongly expressed in the VZ where NSPCs are confined at the embryonic stages. We tried to identify hypoxic areas of developing cortex by using Hypoxyprobe (pimonidazole) [49] that can detect hypoxic tissue visibly by immunohistochemistry, however the signal of Hypoxyprobe was not observed. Since Hypoxyprobe-protein adducts are formed under 10 mmHg of pO_2_, VZ might not be under such conditions at E14. Supporting this observation, signal of Hypoxyprobe can be detected in VZ at E12 or E13.5 but not at E14 [25, 26]. Based on Hif-1a expression, hypoxic conditions of VZ at E14 is relatively mild and Hypoxyprobe may not recognize such conditions, but the hypoxic condition in VZ at E14 is enough to stabilize Hif-1a. Therefore, hypoxic conditions stabilize Hif-1a and it induces confined expression of VEGF-A in the VZ. Consistently, VEGF and VEGFR2 are highly expressed in the VZ in human embryonic brain [50, 51], suggesting that VEGF-A derived from NSPCs effectively contribute to maintenance of NSPC themselves. Besides, Hif-1a is also stabilized on the apical side of cortex, i.e. VZ, in the midgestation (Fig. 6C). Strong expression of Hif-1a in meninges probably reflect local hypoxia in the developing cortex [52]. Deficiency of neuron-derived VEGF-A impairs brain formation including vasculature after neonate [53], suggesting that neuronal VEGF-A contributes to cortical and hippocampal development. Considering that VEGF-A derived from NSPCs is secreted to the vicinity of themselves as targets, it is reasonable to think that such VEGF-A predominantly contributes to NSPC maintenance. Furthermore, when VEGF-A was conditionally knocked out by nestin promoter-driven Cre recombinase, disruption of VZ was observed due to severe defect of vasculature [52]. In that study (ref. 52), neuronal degeneration is observed in the VEGF-A conditional KO cortex. However it is difficult to distinguish direct or indirect (via vascular formation) effects on NSPCs and neurons. Our current in vitro study strongly indicates that VEGF-A secretion from NSPCs is induced by hypoxic conditions in the VZ of developing brain and such VEGF-A directly acts on NSPC maintenance in an autocrine fashion, thus constructing the NSPC niche by themselves. In addition, VEGF-A derived from NSPCs as well as neurons possibly contributes to formation of vascularization in the VZ at the developing stages when vascular network is incomplete.

## Conclusions

In the developing cortex, NSPCs are confined to the VZ and exposed to hypoxic conditions. In our current in vitro study, the hypoxic conditions dramatically increased the formation of neurospheres compared to normoxic conditions and it was partly due to secretion of VEGF-A from NSPCs. These results suggest that NSPCs themselves contribute to NSPC maintenance via VEGF-A in an autocrine manner under hypoxic conditions at the embryonic stages. In other words, NSPCs themselves are one of the crucial players in the NSPC niche in the developing brain in which hypoxic conditions are formed in the VZ owing to insufficient vasculature and due to immaturity of vascular and glial cells composing NSPC niche. Although unidentified autocrine factors in addition to VEGF-A are remained to be revealed, we at least in part uncovered the self-organization of hypoxic niche by NSPCs. This study will help to elucidate not only mechanisms underlying not only NSPC maintenance but also vascular formation in the brain at the embryonic stages since VEGF-A is an important factor for vasculature development.

## Supplementary Information

**Additional file 1: Figure S1.** (related to Fig. 1C, E, I, J and L) The individual numbers of neurospheres in each experiment. The vertical axes indicate neurosphere number per one dish. The different marks indicate the different individual NSPC lots. The effects of hypoxia are consistent across experiments. A, related to Fig. 1C, *n* = 5. B, related to Fig. 1E, *n* = 3. C, related to Fig. 1I, *n* = 3. D, related to Fig. 1J, *n* = 4. E, related to Fig 1L, *n* = 3. Mean ± SEM

**Additional file 2: Figure S2.** (related to Fig. 1F) Ratio of NSPCs and neurons to total cells in the 2-day cultures of freshly isolated cortical cells and enriched NSPCs. A, Freshly isolated cortical cells were cultured for 2 days under the normoxic or the hypoxic condition. Nestin^+^ NSPCs and Tuj1^+^ neurons were observed at approximately the same percentage (30%) under either condition. Mean ± SEM, *n* = 3 (The 651 cells in the control and the 655 cells in the hypoxia was counted in total.). B, Four-day enriched NSPCs were cultured for 2 days under the normoxic or the hypoxic condition. The ratio of nestin^+^ NSPCs to the total number of cells under each condition was comparable (approximately 80 %). The percentage of Tuj1^+^ neurons under each condition was also comparable (approximately 3 %). Mean ± SEM, *n* = 3 (The 1080 cells in the control and the 955 cells in the hypoxia was counted in total.)

**Additional file 3:** **Figure S3.** (related to Fig. 1F) GFAP expression in the enriched NSPC culture. The enriched NSPCs were cultured for 2-days in the presence of FGF2 under the normoxia or hypoxia. The cells cultured with LIF (80 ng/ml, ESGRO LIF, Merck ESG1106) and BMP2 (80 ng/ml, R&D Systems, 355-BM) under the normoxia was used as positive control for GFAP expression. Scale bar = 100 μm

**Additional file 4:** **Figure S4.** Classification of neurosphere diameter (related to Figure 1J). The neurospheres were formed from the enriched NSPCs derived from E14 cerebral cortex. The 1,489 neurospheres under the normoxia and the 4,474 neurospheres under the hypoxia were measured. (A) Neurospheres with diameters smaller than 50 μm and those with diameters of 50 μm or larger were counted separately. (B) The ratio of neurospheres with diameters smaller than 50 μm and those with diameters of 50 μm or larger

**Additional file 5: Figure S5.** Expression of markers for NSPC and neurons in the NSPC culture. The enriched NSPCs derived from E14 cerebral cortex were cultured for one day in the N2 and FGF2 supplemented medium. Then the cells were cultured for 4 days under normoxia or hypoxia. NeuN^+^ neurons were significantly decreased by the hypoxic condition (Mean ± SEM, *n* = 4, *** *p* = 0.00028. The 3,641 cells in normoxia group and the 3,760 cells in hypoxia group were counted in total). Scale bar = 100 μm

**Additional file 6:** **Figure S6.** (related to Fig. 2B) Representative microphotographs of neurospheres derived from enriched E14.5 NSPCs. NSPCs were cultured under the conditioned medium collected from normoxic- or hypoxic cortical cell culture. Scale bar = 200 μm

**Additional file 7: Figure S7.** (related to Fig. 2B and E) Neurosphere number in each samples. The vertical axes indicate neurosphere number per one dish. The different marks indicate the different individual NSPC lots. A, related to Fig. 2B, *n* = 4. B, related to Fig. 2E, *n* = 3. Mean ± SEM

**Additional file 8: Figure S8.** (related to Fig. 2B) Neurosphere formation from enriched E14.5 NSPCs under the conditioned medium prepared from the absence cell culture. The medium component was not affected by hypoxic condition regarding neurosphere formation. Mean ± SEM, *n* = 3

**Additional file 9: Figure S9.** (related to Fig. 3A, B, C, H and I) The number of neurospheres in each samples. The vertical axes indicate neurosphere number per one dish. The different marks indicate the different individual NSPC lots. A, related to Fig. 3A, *n* = 3. B, related to Fig. 3B, *n* = 4. C, related to Fig. 3C, *n* = 3. D, related to Fig. 3H, *n* = 4. E, related to Fig. 3I, *n* = 4. Mean ± SEM

**Additional file 10: Figure S10.** (related to Fig. 3A) Quantification of neurospheres from E11.5 NSPCs by administration of VEGF-A. Neurosphere formation from enriched E11.5 NSPCs was enhanced by exogenous VEGF-A like as the case of E14.5 NSPCs. Mean ± SEM, *n* = 3, * *p* = 0.017 by one-sided test, *p* = 0.059 by two-sided test

**Additional file 11: Figure S11.** (related to Fig. 3B) Effect of SU1498 on neurosphere formation in the presence of VEGF-A. Mean ± SEM, *n* = 4 in 0, 0.35, and 0.7 μM of SU1498; *n* = 3 in 1.4 and 2.8 μM of SU1498; *n* = 2 in 0.175 μM of SU1498; ^a^ *p* = 0.0055, ^b^ *p* = 0.063, ^c^ *p* = 0.0070, ^d^ *p* = 0.0027, ^e^ *p* = 0.015, ^f^ *p* = 0.043

**Additional file 12:** **Figure S12.** Full-length gel images of Fig. 3D, F. A. Full-length gel image of Fig. 3D. B, C. Full-length gel image of Fig. 3F. The yellow dotted squares indicate the cropped area. The VEGF-A gel image of B was inverted horizontally in Fig. 3F. M, 1 kb Plus DNA Ladder (New England BioLabs, N3200); N, normoxia; H, hypoxia

**Additional file 13: Figure S13.** (related to Fig. 3E and G) VEGF-A secretion in the NSPC culture medium. *n* = 4 (E11.5, Fig. 3E), *n* = 3 (E14.5, Fig. 3G), mean ± SEM

**Additional file 14: Figure S14.** (related to Fig. 4A and B) Neurosphere number in each samples. The vertical axes indicate neurosphere number per one dish. The different marks indicate the different individual NSPC lots. A, related to Fig. 4A, *n* = 3. B, related to Fig. 4B, *n* = 3. Mean ± SEM

**Additional file 15: Figure S15.** (related to Fig. 5A and B) NSPC or neuronal marker expression in the apoptotic cells. The active caspase-3^+^ cells were counted in the experiments described in Fig S5. Scale bar = 100 μm. Mean ± SEM, *n* = 4, ^a^ *p* = 0.0091, ^b^ *p* = 0.0034, ^c^ *p* = 0.37, ^d^ *p* = 0.65

## Data Availability

The datasets are available from the corresponding author upon reasonable request.

## Abbreviations

NSPCs: neural stem/progenitor cells
VEGF: vascular endothelial growth factor
Hif: hypoxia inducible factor
SVZ: subventricular zone
LV: lateral ventricle
VZ: ventricular zone
SGZ: subgranular zone
FGF2: fibroblast growth factor 2
CM: conditioned medium
GFAP: glial fibrillary acidic protein
LIF: leukemia inhibitory factor
BMP2: bone morphogenetic protein 2
VEGFR: VEGF receptor
