# Supplementary figures and images for: Organization of self-advantageous niche by neural stem/progenitor cells during development via autocrine VEGF-A under hypoxia
Source: Inflamm Regen. 2023 Feb 1;43:8. doi: 10.1186/s41232-022-00254-2 (PMC9893632; doi:10.1186/s41232-022-00254-2)

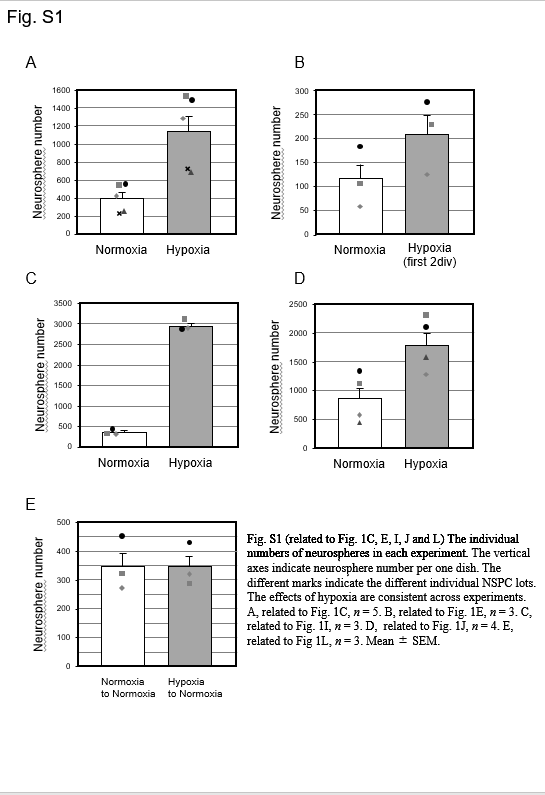

Supplement: Supplementary file 1 — Additional file 1: Figure S1. (related to Fig. 1C, E, I, J and L) The individual numbers of neurospheres in each experiment. The vertical axes indicate neurosphere number per one dish. The different marks indicate the different individual NSPC lots. The effects of hypoxia are consistent across experiments. A, related to Fig. 1C, n = 5. B, related to Fig. 1E, n = 3. C, related to Fig. 1I, n = 3. D, related to Fig. 1J, n = 4. E, related to Fig 1L, n = 3. Mean ± SEM [file 41232_2022_254_MOESM1_ESM.png]

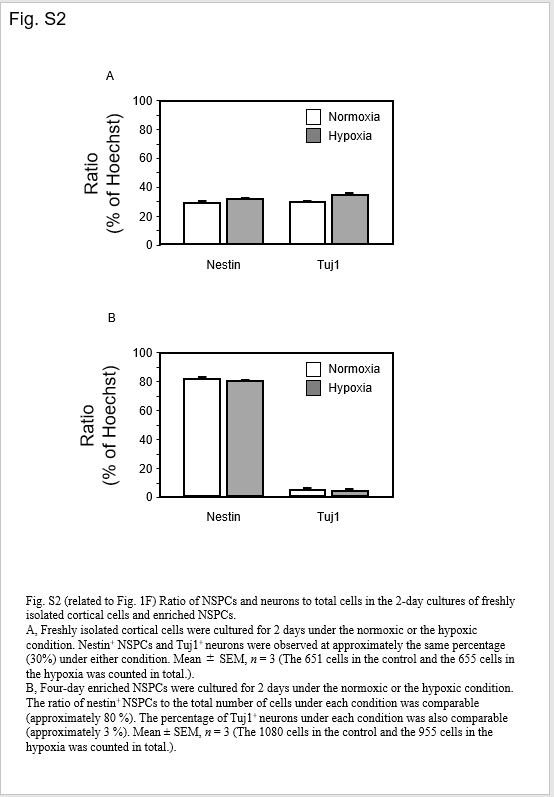

Supplement: Supplementary file 2 — Additional file 2: Figure S2. (related to Fig. 1F) Ratio of NSPCs and neurons to total cells in the 2-day cultures of freshly isolated cortical cells and enriched NSPCs. A, Freshly isolated cortical cells were cultured for 2 days under the normoxic or the hypoxic condition. Nestin+ NSPCs and Tuj1+ neurons were observed at approximately the same percentage (30%) under either condition. Mean ± SEM, n = 3 (The 651 cells in the control and the 655 cells in the hypoxia was counted in total.). B, Four-day enriched NSPCs were cultured for 2 days under the normoxic or the hypoxic condition. The ratio of nestin+ NSPCs to the total number of cells under each condition was comparable (approximately 80 %). The percentage of Tuj1+ neurons under each condition was also comparable (approximately 3 %). Mean ± SEM, n = 3 (The 1080 cells in the control and the 955 cells in the hypoxia was counted in total.) [file 41232_2022_254_MOESM2_ESM.png]

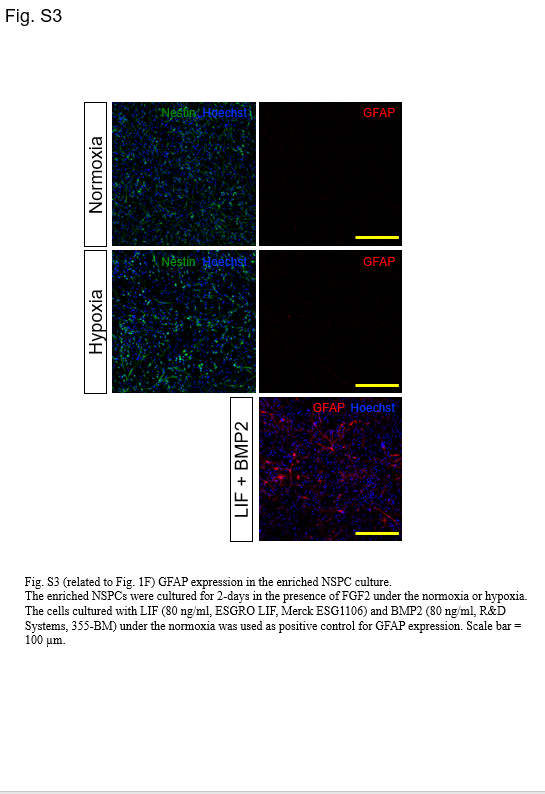

Supplement: Supplementary file 3 — Additional file 3: Figure S3. (related to Fig. 1F) GFAP expression in the enriched NSPC culture. The enriched NSPCs were cultured for 2-days in the presence of FGF2 under the normoxia or hypoxia. The cells cultured with LIF (80 ng/ml, ESGRO LIF, Merck ESG1106) and BMP2 (80 ng/ml, R&D Systems, 355-BM) under the normoxia was used as positive control for GFAP expression. Scale bar = 100 μm [file 41232_2022_254_MOESM3_ESM.png]

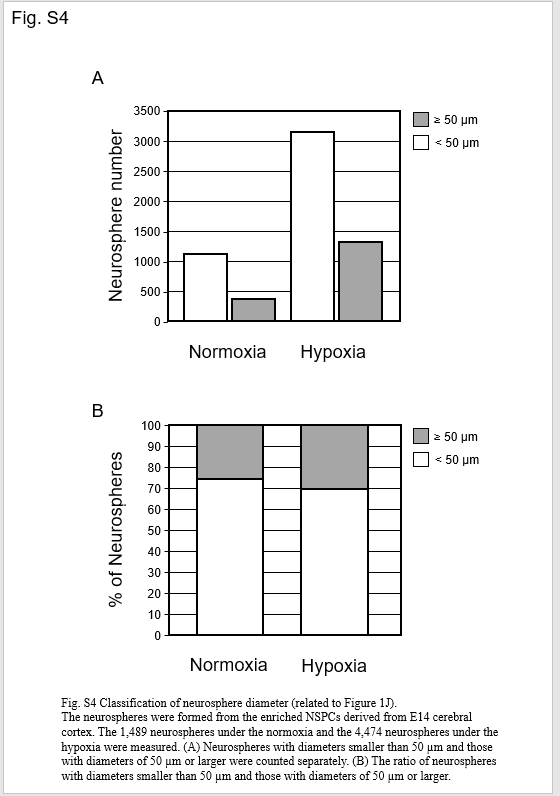

Supplement: Supplementary file 4 — Additional file 4: Figure S4. Classification of neurosphere diameter (related to Figure 1J). The neurospheres were formed from the enriched NSPCs derived from E14 cerebral cortex. The 1,489 neurospheres under the normoxia and the 4,474 neurospheres under the hypoxia were measured. (A) Neurospheres with diameters smaller than 50 μm and those with diameters of 50 μm or larger were counted separately. (B) The ratio of neurospheres with diameters smaller than 50 μm and those with diameters of 50 μm or larger [file 41232_2022_254_MOESM4_ESM.png]

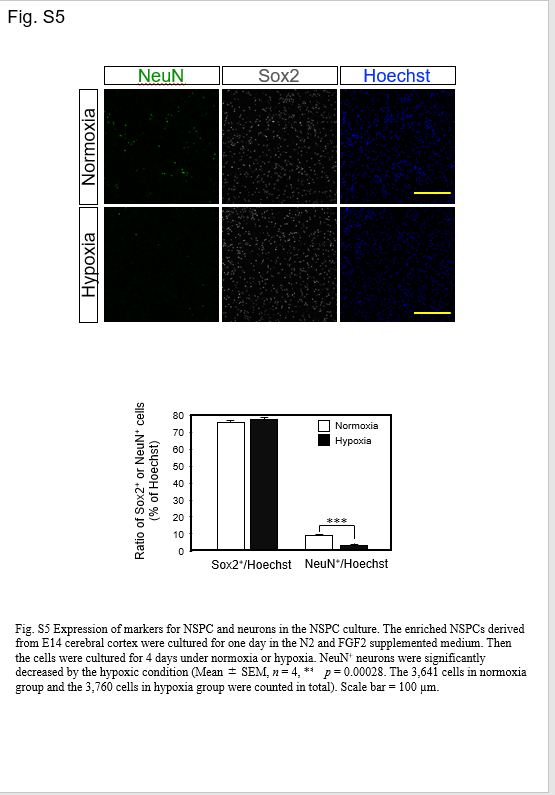

Supplement: Supplementary file 5 — Additional file 5: Figure S5. Expression of markers for NSPC and neurons in the NSPC culture. The enriched NSPCs derived from E14 cerebral cortex were cultured for one day in the N2 and FGF2 supplemented medium. Then the cells were cultured for 4 days under normoxia or hypoxia. NeuN+ neurons were significantly decreased by the hypoxic condition (Mean ± SEM, n = 4, *** p = 0.00028. The 3,641 cells in normoxia group and the 3,760 cells in hypoxia group were counted in total). Scale bar = 100 μm [file 41232_2022_254_MOESM5_ESM.png]

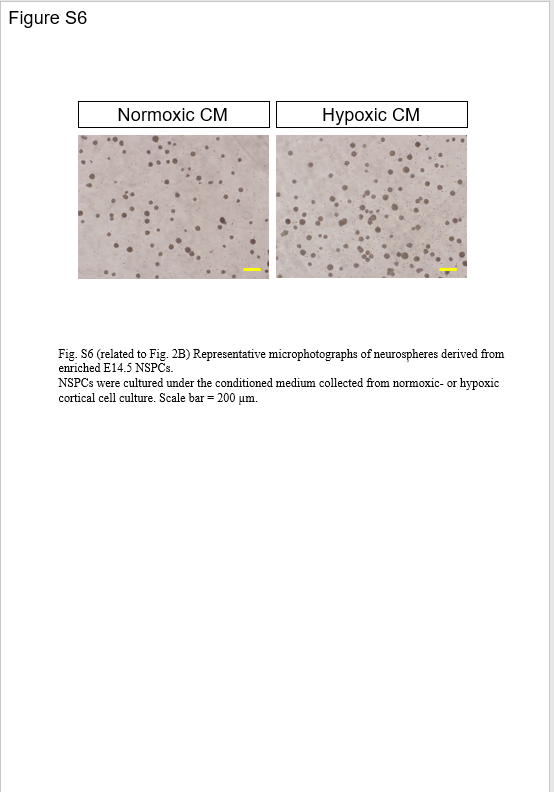

Supplement: Supplementary file 6 — Additional file 6: Figure S6. (related to Fig. 2B) Representative microphotographs of neurospheres derived from enriched E14.5 NSPCs. NSPCs were cultured under the conditioned medium collected from normoxic- or hypoxic cortical cell culture. Scale bar = 200 μm [file 41232_2022_254_MOESM6_ESM.png]

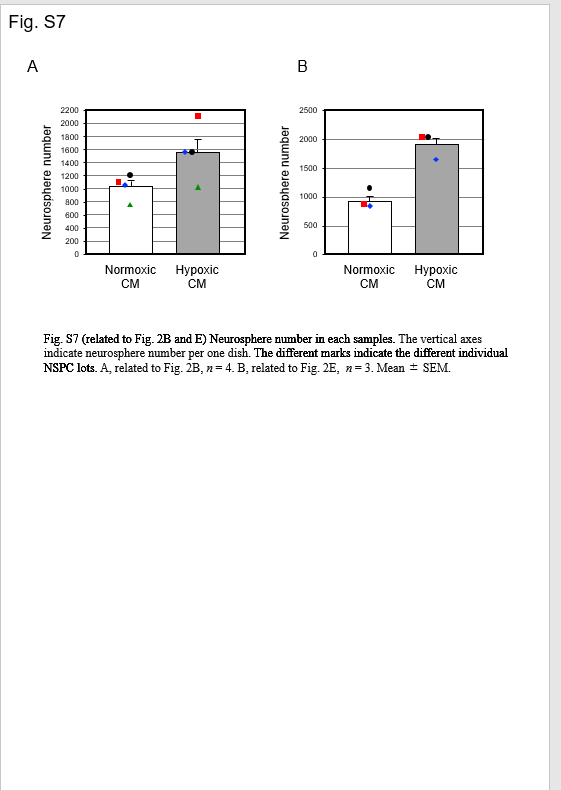

Supplement: Supplementary file 7 — Additional file 7: Figure S7. (related to Fig. 2B and E) Neurosphere number in each samples. The vertical axes indicate neurosphere number per one dish. The different marks indicate the different individual NSPC lots. A, related to Fig. 2B, n = 4. B, related to Fig. 2E, n = 3. Mean ± SEM [file 41232_2022_254_MOESM7_ESM.png]

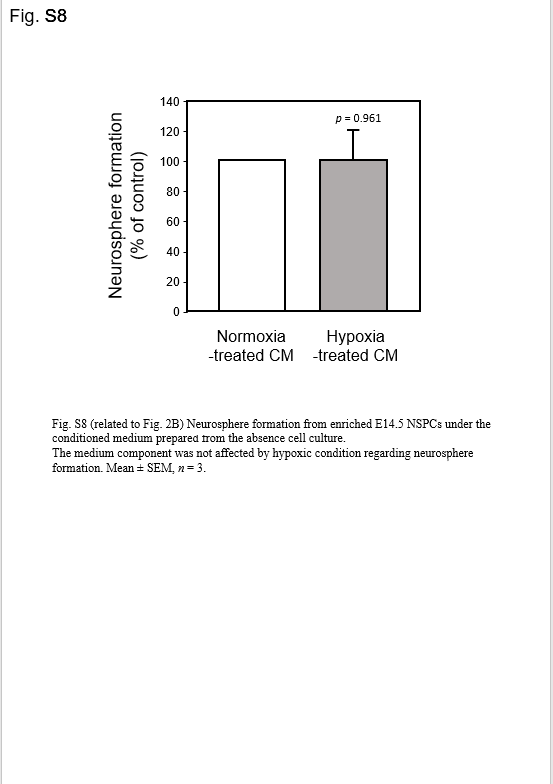

Supplement: Supplementary file 8 — Additional file 8: Figure S8. (related to Fig. 2B) Neurosphere formation from enriched E14.5 NSPCs under the conditioned medium prepared from the absence cell culture. The medium component was not affected by hypoxic condition regarding neurosphere formation. Mean ± SEM, n = 3 [file 41232_2022_254_MOESM8_ESM.png]

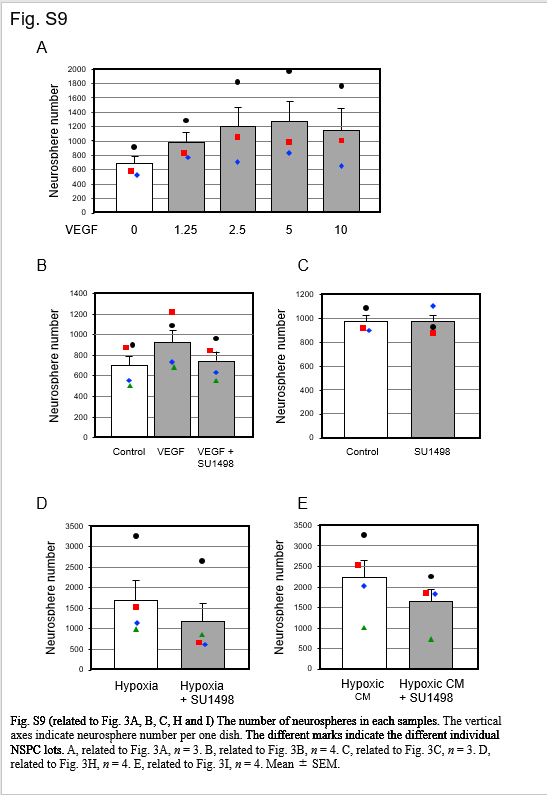

Supplement: Supplementary file 9 — Additional file 9: Figure S9. (related to Fig. 3A, B, C, H and I) The number of neurospheres in each samples. The vertical axes indicate neurosphere number per one dish. The different marks indicate the different individual NSPC lots. A, related to Fig. 3A, n = 3. B, related to Fig. 3B, n = 4. C, related to Fig. 3C, n = 3. D, related to Fig. 3H, n = 4. E, related to Fig. 3I, n = 4. Mean ± SEM [file 41232_2022_254_MOESM9_ESM.png]

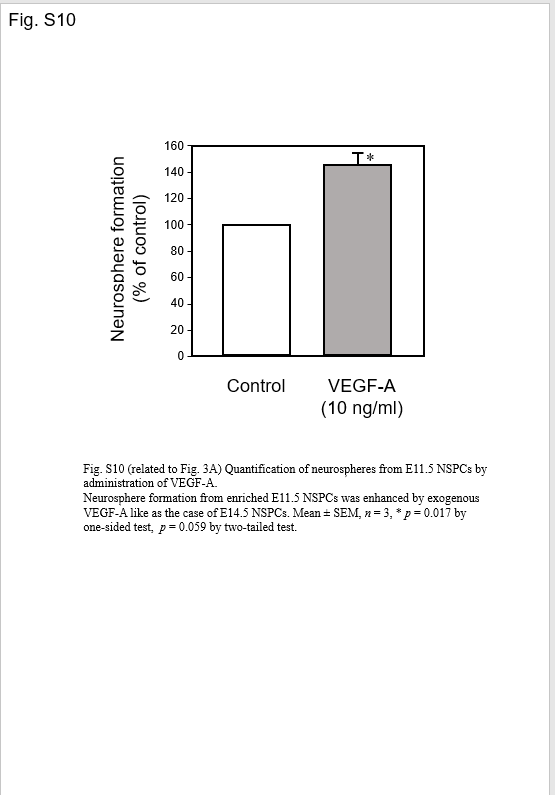

Supplement: Supplementary file 10 — Additional file 10: Figure S10. (related to Fig. 3A) Quantification of neurospheres from E11.5 NSPCs by administration of VEGF-A. Neurosphere formation from enriched E11.5 NSPCs was enhanced by exogenous VEGF-A like as the case of E14.5 NSPCs. Mean ± SEM, n = 3, * p = 0.017 by one-sided test, p = 0.059 by two-sided test [file 41232_2022_254_MOESM10_ESM.png]

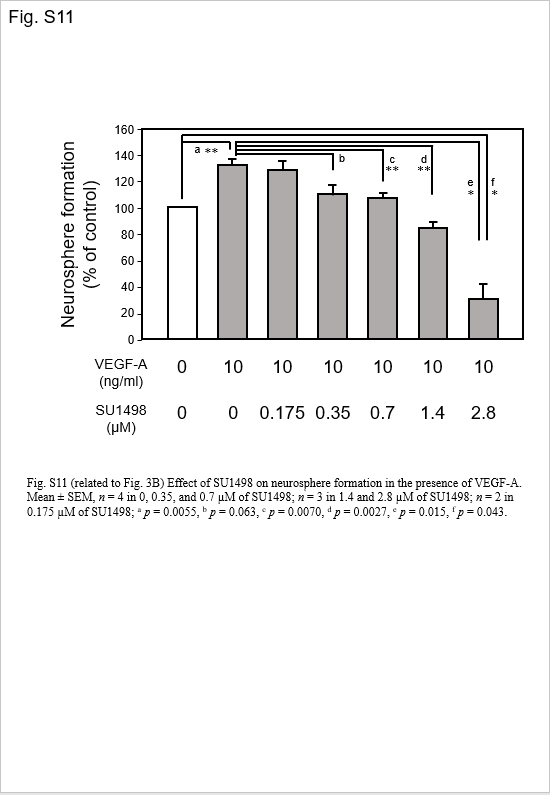

Supplement: Supplementary file 11 — Additional file 11: Figure S11. (related to Fig. 3B) Effect of SU1498 on neurosphere formation in the presence of VEGF-A. Mean ± SEM, n = 4 in 0, 0.35, and 0.7 μM of SU1498; n = 3 in 1.4 and 2.8 μM of SU1498; n = 2 in 0.175 μM of SU1498; a p = 0.0055, b p = 0.063, c p = 0.0070, d p = 0.0027, e p = 0.015, f p = 0.043 [file 41232_2022_254_MOESM11_ESM.png]

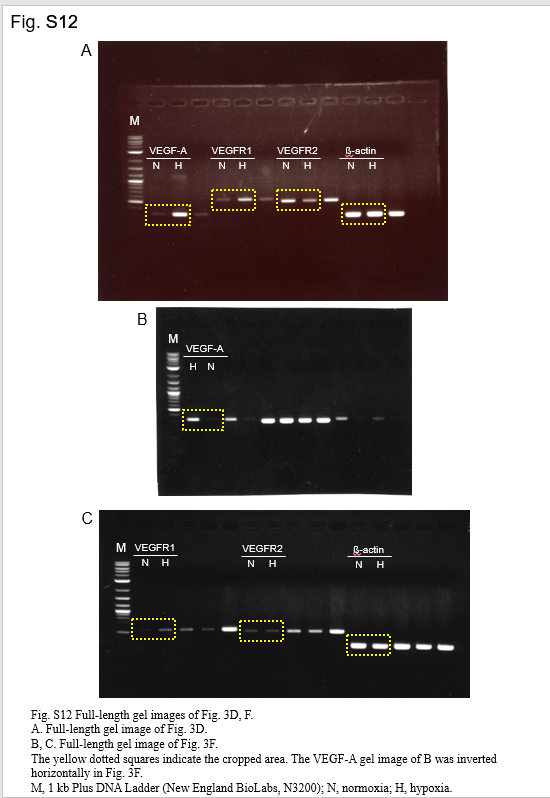

Supplement: Supplementary file 12 — Additional file 12: Figure S12. Full-length gel images of Fig. 3D, F. A. Full-length gel image of Fig. 3D. B, C. Full-length gel image of Fig. 3F. The yellow dotted squares indicate the cropped area. The VEGF-A gel image of B was inverted horizontally in Fig. 3F. M, 1 kb Plus DNA Ladder (New England BioLabs, N3200); N, normoxia; H, hypoxia [file 41232_2022_254_MOESM12_ESM.png]

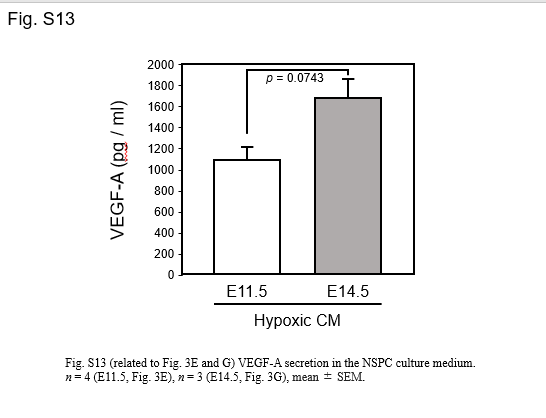

Supplement: Supplementary file 13 — Additional file 13: Figure S13. (related to Fig. 3E and G) VEGF-A secretion in the NSPC culture medium. n = 4 (E11.5, Fig. 3E), n = 3 (E14.5, Fig. 3G), mean ± SEM [file 41232_2022_254_MOESM13_ESM.png]

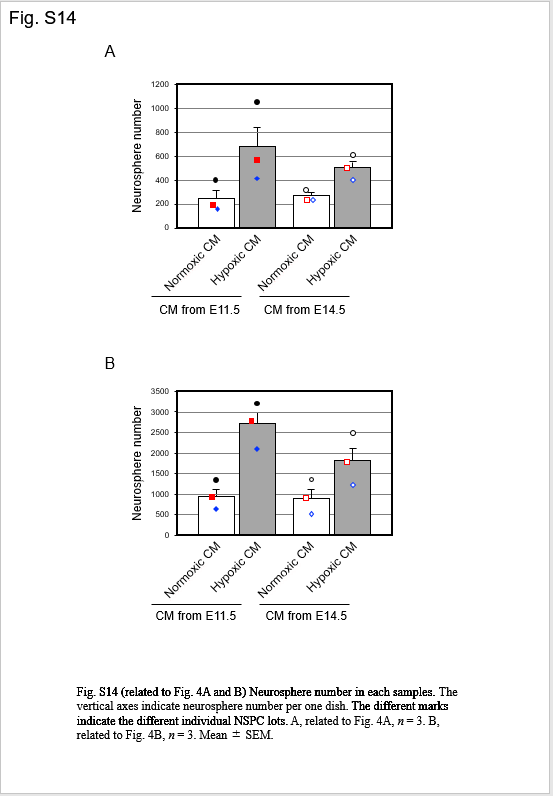

Supplement: Supplementary file 14 — Additional file 14: Figure S14. (related to Fig. 4A and B) Neurosphere number in each samples. The vertical axes indicate neurosphere number per one dish. The different marks indicate the different individual NSPC lots. A, related to Fig. 4A, n = 3. B, related to Fig. 4B, n = 3. Mean ± SEM [file 41232_2022_254_MOESM14_ESM.png]

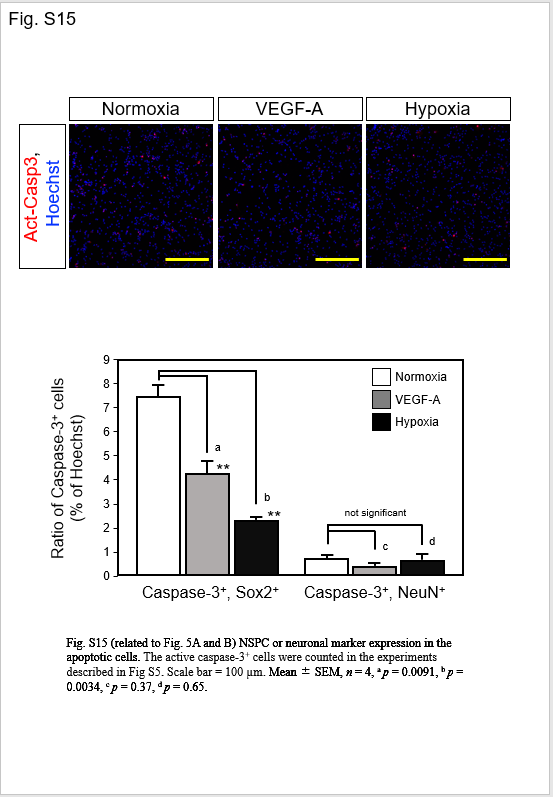

Supplement: Supplementary file 15 — Additional file 15: Figure S15. (related to Fig. 5A and B) NSPC or neuronal marker expression in the apoptotic cells. The active caspase-3+ cells were counted in the experiments described in Fig S5. Scale bar = 100 μm. Mean ± SEM, n = 4, a p = 0.0091, b p = 0.0034, c p = 0.37, d p = 0.65 [file 41232_2022_254_MOESM15_ESM.png]
